# NEEMP: software for validation, accurate calculation and fast parameterization of EEM charges

**DOI:** 10.1186/s13321-016-0171-1

**Published:** 2016-10-17

**Authors:** Tomáš Raček, Jana Pazúriková, Radka Svobodová Vařeková, Stanislav Geidl, Aleš Křenek, Francesco Luca Falginella, Vladimír Horský, Václav Hejret, Jaroslav Koča

**Affiliations:** 1 CEITEC – Central European Institute of Technology, Masaryk University Brno, Kamenice 5, 625 00 Brno, Czech Republic; 2 National Centre for Biomolecular Research, Faculty of Science, Masaryk University Brno, Kamenice 5, 625 00 Brno, Czech Republic; 3 Faculty of Informatics, Masaryk University Brno, Botanická 68a, 602 00 Brno, Czech Republic; 4 Institute of Computer Science, Masaryk University Brno, Botanická 68a, 602 00 Brno, Czech Republic

**Keywords:** Partial atomic charges, Electronegativity equalization method, EEM, EEM parameterization, wwPDB CCD database

## Abstract

**Background:**

The concept of partial atomic charges was first applied in physical and organic chemistry and was later also adopted in computational chemistry, bioinformatics and chemoinformatics. The electronegativity equalization method (EEM) is the most frequently used approach for calculating partial atomic charges. EEM is fast and its accuracy is comparable to the quantum mechanical charge calculation method for which it was parameterized. Several EEM parameter sets for various types of molecules and QM charge calculation approaches have been published and new ones are still needed and produced. Methodologies for EEM parameterization have been described in a few articles, but a software tool for EEM parameterization and EEM parameter sets validation has not been available until now.

**Results:**

We provide the software tool NEEMP (http://ncbr.muni.cz/NEEMP), which offers three main functionalities: EEM parameterization [via linear regression (LR) and differential evolution with local minimization (DE-MIN)]; EEM parameter set validation (i.e., validation of coverage and quality) and EEM charge calculation. NEEMP functionality is shown using a parameterization and a validation case study. The parameterization case study demonstrated that LR is an appropriate approach for smaller and homogeneous datasets and DE-MIN is a suitable solution for larger and heterogeneous datasets. The validation case study showed that EEM parameter set coverage and quality can still be problematic. Therefore, it makes sense to verify the coverage and quality of EEM parameter sets before their use, and NEEMP is an appropriate tool for such verification. Moreover, it seems from both case studies that new EEM parameterizations need to be performed and new EEM parameter sets obtained with high quality and coverage for key structural databases.

**Conclusion:**

We provide the software tool NEEMP, which is to the best of our knowledge the only available software package that enables EEM parameterization and EEM parameter set validation. Additionally, its DE-MIN parameterization method is an innovative approach, developed by ourselves and first published in this work. In addition, we also prepared four high-quality EEM parameter sets tailored to ligand molecules.

**Graphical abstract:**
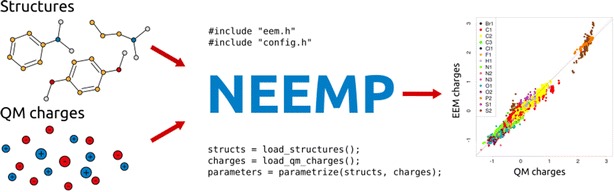
.

**Electronic supplementary material:**

The online version of this article (doi:10.1186/s13321-016-0171-1) contains supplementary material, which is available to authorized users.

## Background

Information about electron density distribution in a molecule is very useful, because it gives us an insight into the chemical behavior of the molecule and helps us to understand its reactivity. We can express this information via the electron populations of orbitals. But this approach is highly complex, resource-demanding and inconvenient for applications. A markedly more efficient solution is to summarize the electron density “belonging” to each atom into one overall number—partial atomic charge. The concept of partial atomic charges was first applied in physical and organic chemistry, and because of its usefulness and intuitiveness it was also adopted in computational chemistry (e.g., docking [1], conformers generation [2] or molecular dynamics [3, 4]), bioinformatics (e.g., similarity searches [5, 6], molecular structure comparison [7, 8]) and chemoinformatics (e.g., QSAR and QSPR modelling [9–14], pharmacophore design [15], virtual screening [16]).

The most common and also the most accurate charge calculation method is the application of quantum mechanics (QM). Specifically, QM is employed for calculating electron orbital populations, and the populations are divided among the individual atoms using a charge calculation scheme. Unfortunately, there is no one universal and best method for QM charge calculation. We can use various combinations of QM theory level and basis set to obtain information about electron distribution in the orbitals. In addition, we can also apply different charge calculation schemes to process this information and obtain a sum of electron density for each individual atom. Well-known charge calculation schemes are for example Mulliken population analysis (MPA) [17, 18], Natural population analysis (NPA) [19, 20], the atoms-in-molecules (AIM) approach [21, 22], CHELPG [23] and Merz-Singh-Kollman (MK) [24, 25] method. Therefore, many various combinations of QM theory level, basis set and charge calculation schemes can be used for QM charge calculation. Different combinations are suitable for different types of applications.

Although a wide spectrum of QM charge calculation methods are available, all the methods have a major limitation—they are very time-demanding. For this reason, empirical charge calculation approaches have been developed [3, 26–33]. One of the most frequently used empirical approaches is the electronegativity equalization method (EEM). It is based on DFT and it calculates the charges via the following equation set:

1 $$\begin{aligned} \begin{bmatrix} B_{1}&\quad \frac{\kappa }{R_{1,2}}&\quad \cdots&\quad \frac{\kappa }{R_{1,N}}&\quad -1\\ \frac{\kappa }{R_{2,1}}&\quad B_{2}&\quad \cdots&\quad \frac{\kappa }{R_{2,N}}&\quad -1 \\ \vdots&\quad \vdots&\quad \ddots&\quad \vdots&\quad \vdots \\ \frac{\kappa }{R_{N,1}}&\quad \frac{\kappa }{R_{N,2}}&\quad \cdots&\quad B_{N}&\quad -1\\ 1&\quad 1&\quad \cdots&\quad 1&\quad 0&\quad \end{bmatrix}.\begin{bmatrix}q_{1}\\ q_{2}\\ \vdots \\ q_{N}\\ \bar{\chi } \\\end{bmatrix}= \begin{bmatrix}-A_{1}\\ -A_{2}\\ \vdots \\ -A_{N}\\ Q\\ \end{bmatrix} \end{aligned}$$

where $q_i$ is the charge of an atom *i*; $R_{i,j}$ is the distance between atoms *i* and *j*; *Q* is the total charge of the molecule; *N* is the number of atoms in the molecule; $\overline{\chi }$ is the molecular electronegativity, and $A_i$, $B_i$ and $\kappa$ are empirical parameters. The parameters $A_i$ and $B_i$ vary for individual atom types, where atom type is a combination of element type and maximal bond order of the atom *i*. For example, the atom type N3 means that the atom is nitrogen and it creates at least one triple bond with its neighbors.

The main advantages of EEM are the following: It provides conformationally dependent charges (i.e., charges sensitive to conformational change), it has low time complexity (i.e., $\theta (N^3)$) and its accuracy is comparable to QM approaches. A limitation of EEM is that it requires a set of empirical parameters (i.e., $A_i$ and $B_i$ and $\kappa$). These empirical parameters are calculated from QM charges using a process of EEM parameterization. Consequently, EEM can mimic the QM charge calculation approach for which it was parameterized. In addition, because the EEM parameter set is calculated for a specific dataset of molecules, it provides the highest quality of charges on molecules similar to this dataset. Therefore, the EEM parameterizations are often performed for different QM charge calculation approaches and also for various types of molecules (small organic molecules, peptides, proteins, ligands, organometals etc.) to achieve the best accuracy of EEM charges. A lot of EEM parameter sets were published in the past [34–39] and new EEM parameter sets are still in development [40]. Unfortunately, the EEM parameter sets published in the past often only contain parameters for a few atom types and therefore cannot be used for molecules including other atoms.

Because of the strong demand for EEM parameterization, several EEM parameterization approaches were developed. The most widely known is an application of linear regression (LR), described by [31] and [35] and utilized for the preparation of many EEM parameter sets, e.g., in [34–37, 39, 40]. An alternative approach is differential evolution, described and used in [38]. Also other approaches (e.g., accelerated random search [38], particle swarm optimization algorithm [38]) were tested for EEM parameterization, but they were not applicable. Unfortunately, no software is currently available for EEM parameterization or for the validation of EEM parameters. All the software tools related to EEM (e.g., OpenBabel [41], Balloon [42], EEM Solver [43]) are focused purely on EEM charge calculation.

This motivated us to create such a tool and to provide it to the research community. Specifically, we developed NEEMP—a software for fast EEM parameterization, EEM parameters validation and also EEM charge calculation. NEEMP offers two approaches for EEM parameterization—the standard LR method and differential evolution with local minimization (DE-MIN) approach, recently developed by ourselves. NEEMP also provides two validation modes—a validation of EEM charge quality and coverage. The quality validation compares EEM charges with relevant QM charges and reports common correlation coefficients. The coverage validation analyzes how large a proportion of the molecules from the input database can be processed using the validated EEM parameter set (therefore the validated EEM parameter set covers these molecules).

NEEMP is available here: http://ncbr.muni.cz/NEEMP, source codes are also in (Additional file 1). NEEMP is also documented in Bio.Tools [57]—a portal of bioinformatics resources world-wide.

NEEMP performance was demonstrated via two case studies—the first was focused on EEM parameterization and the second on EEM parameter validation. In both case studies, we worked with molecules from the databases which are very interesting and important for the life science community. Specifically, the wwPDB CCD database [44] of all ligands present in biomacromolecular structures, the DrugBank database [45] of drug compounds and the PubChem database [46], containing a huge amount of organic molecules.

## Description of the tool

NEEMP offers the user three modes—calculation, parameterization and validation mode.

### Calculation mode

 In this mode, NEEMP calculates EEM charges for the input molecule(s) using a user-defined EEM parameter set. Therefore, this mode requires 3D structure(s) of the input molecule(s), information about their total charge (0 for neutral molecules, nonzero real number for charged molecules) and the input EEM parameter set. The charge calculation is performed using Eq. (1) and the values of EEM charges are returned.

### Parameterization mode

 This mode is for calculating EEM parameters. An input for this calculation is a training set of molecules (i.e., their 3D structures) and QM charges for each molecule. NEEMP can calculate EEM parameters for neutral molecules and also for ions. The parameterization can be performed via two approaches: LR and DE-MIN.

The LR approach is implemented according to its description in [35]. The only extension is that the previous implementation only enables the best performing EEM parameter set to be selected via searching for the highest squared Pearson coefficient ($R^2$). NEEMP also offers a selection based on the lowest average atom type root mean square difference ($avg(RMSD_a)$). The $avg(RMSD_a)$ is calculated as an average of root mean square difference values for individual atom types ($RMSD_a$). For simplification, the $avg(RMSD_a)$ metrics will be abbreviated below as “RMSD metrics”. Optionally, the program can also attempt to discard some of the molecules in the training set, which may yield better results in some situations.

The DE-MIN algorithm is one that we recently developed ourselves. Advanced EEM parameterization approaches [38] usually combine global optimization methods (evolution algorithms, genetic algorithms, simulated annealing) with local optimization methods (simplex method, conjugated gradients or other). These advanced approaches search for the set of EEM parameters that fit QM charges from the training set in the best possible way. They offer a more robust approach than LR, therefore they are applicable even for the heterogeneous training set. We combined differential evolution (DE) with local minimization, which has not been done before. DE starts with generating a random population of vectors, each vector consisting of $\kappa$, $A_i$ and $B_i$ for all atom types. Afterwards, all vectors (i.e., EEM parameter sets) are evaluated: EEM charges are computed using the parameter set and compared to QM charges via the chosen metrics ($R^2$, *RMSD*). Vectors with at least slightly promising results (e.g., $R^2 > 0.2$ and $R > 0$) are minimized by the local minimization method NEWUOA [47]. This step significantly increases the quality of population vectors. Then evolution is mimicked over many iterations: a new vector is created as a combination of two vectors randomly selected from the population. Again, if the vector is promising, we apply local minimization. The best vector found during the evolution iterations is polished again via a few more iterations of NEWUOA and presented as the result. Because of the random generation of the vectors, the DE-MIN approach works stochastically, i.e., even for identical inputs, the results will slightly differ.

### Validation mode

 This mode enables us to perform two types of EEM parameter set validation—coverage validation and quality validation.

The coverage validation analyzes, how large a proportion of the molecules from the input database are composed only of the atom types included in the input EEM parameter set. This means they are “covered” by this EEM parameter set. Therefore, the coverage validation requires an input database (containing the 3D structures of molecules) and the validated EEM parameters. This validation returns a count and a percentage of molecules from the database which are covered by the parameters. Additionally, it identifies which particular molecules are covered and which are not.

**Fig. 1:**
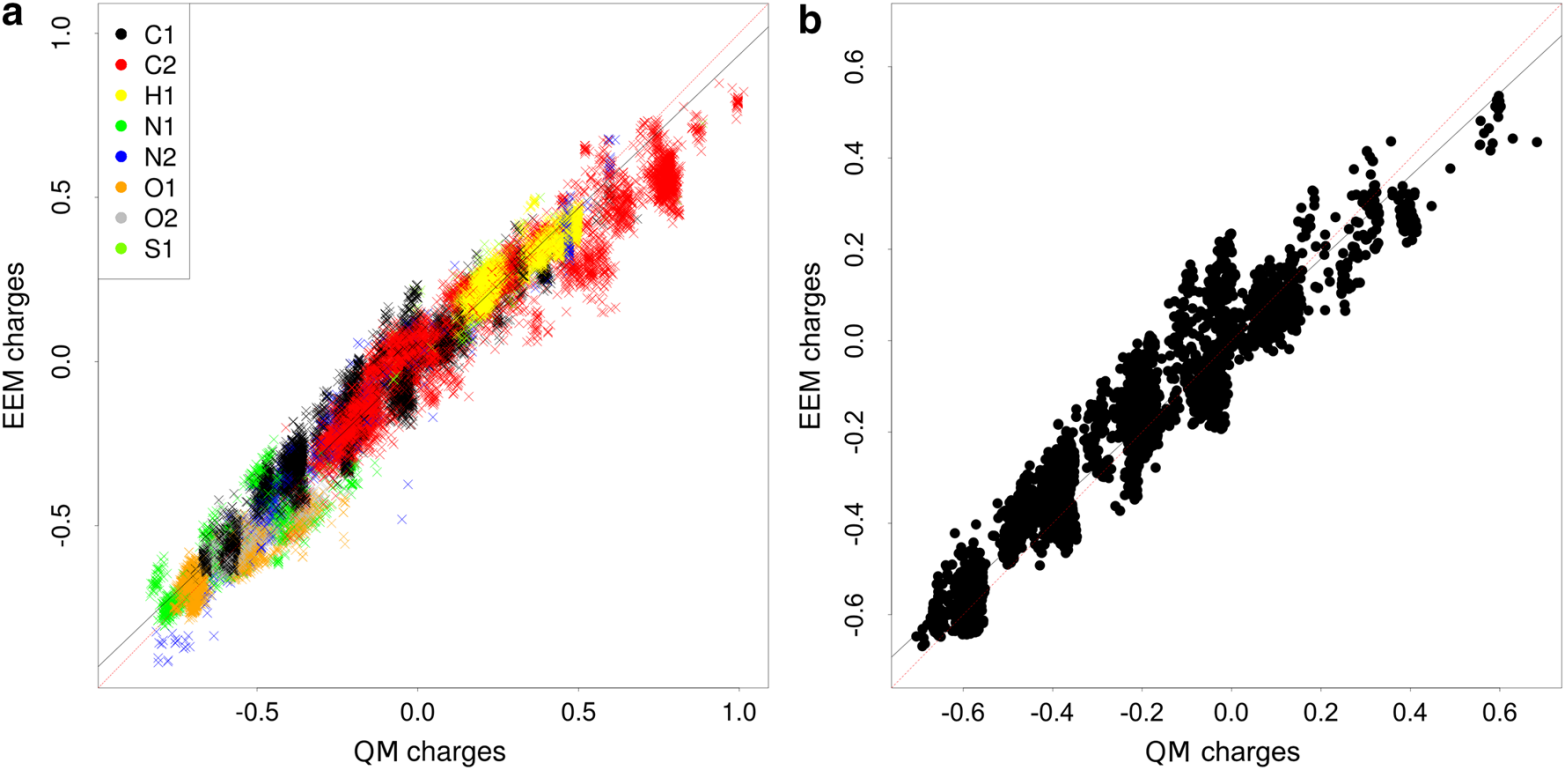
Example of quality validation outputs–graphs of correlation between reference charges and EEM charges. Correlation graph for all atoms (**a**) and correlation graph for C1 atom type (**b**)

The quality validation tests the accuracy of the EEM charges produced by the input EEM parameter set on the validated dataset. Therefore, the inputs are the validated EEM parameters, the dataset (3D structures of molecules) and relevant QM charges for each molecule. This validation of quality provides three types of quality criteria—summary criteria (calculated for the whole dataset), atom type criteria (calculated for all the atom types available in the validated EEM parameter set) and criteria for individual molecules. The summary validation criteria are the Pearson coefficient (*R*), the squared Pearson coefficient ($R^2$), the Spearman coefficient, the squared Spearman coefficient, root mean square deviation (*RMSD*), absolute average difference ($\Delta$) and maximal absolute difference ($\Delta _{max}$)). The atom type criteria and the criteria for individual molecules are the same, but they are calculated for all the relevant atom types or molecules, respectively. In addition, NEEMP also generates graphs depicting the correlation between reference charges and EEM charges. Specifically, it creates graphs showing the dependency for all the atoms (see Fig. 1a) and also graphs for individual atomic types (see Fig. 1b).

## Implementation

NEEMP is implemented as a single C program which switches among its three modes (calculation, parameterization, and validation) according to a command line option. Therefore, its distribution is trivial—only a single binary and a few libraries for a particular platform are downloaded. In total, the program size is approximately 5000 lines of code.

The most compute-intensive part (common to all program modes) is the solution of the linear equation system (1). We use LAPACK DSPSV/DSPSVX calls [48] (Cholesky matrix factorization followed by backward substitution and optionally iterative refinement). The LR parameterization method solves another system of linear equations to do the least squares fitting; in this case we use a LAPACK DGELS call (QR factorization) which can handle nearly singular matrices more accurately.

Both open-source and Intel MKL LAPACK implementations are supported.

The DE-MIN parameterization method uses NEWUOA local minimization, the program links to Powell’s implementation in its references [47].

The program utilizes simple, coarse grain parallelism. Using the Open MP programming paradigm, several loops—charge calculation for multiple molecules, evaluation over different $\kappa$ values in the LR method, and minimization of multiple parameter sets in DE-MIN—run in parallel on available CPU cores. Of these, the first provides the best speedup.

The program only supports a single file format (SDF), using its internal file parsing routine, hence does not introduce dependencies on other libraries. Other file formats can be easily converted using 3rd-party tools (e.g., Open Babel).

## Results and discussion

We prepared two case studies to show the functionality and performance of NEEMP. The first case study is focused on EEM parameterization and the second on the validation of EEM parameters.

### Parameterization case study

This case study first compares EEM parameterization approaches (Parameterization comparison case study), then shows the parameterization running times (Parameterization running time case study) and afterwards focuses on EEM parameterization for wwPDB CCD (Parameterization calculation case study).

#### Parameterization comparison case study

*Goal* Comparison of EEM parameterization approaches (LR vs. DE-MIN, $R^2$ metric versus *RMSD* metric) and evaluation of which are the most suitable for which types of data.

*Datasets preparation* In this case study, we used four datasets, which are described in Table 1.

**Table 1:** Description of datasets used in parameterization case study

		Dataset			
Denotation		DTP_small	DTP_large	CCD_gen	CCD_exp
Source database		DTP NCI		wwPDB CCD	
Number of molecules		1956	4475	4443	
Atomic types (elements and bond orders)		C1, C2, O1,O2, N1, N2,H, S1	H1, C1, C2,C3, N1, N2,N3, O1, O2,F1, P1, P2,S1, S2, Cl1,Br1, I1	H1, C1, C2, C3, N1,N2, N3, O1, O2, F1,P2, S1, S2, Cl1, Br1	
Size of molecules		6-176 atoms	5-124 atoms	3-305 atoms	
Type of molecules		Small organic molecules	Small organic molecules	Small organic and inorganic molecules, organometals, peptides	
Source of 3D structures		Generated by CORINA			Experimental structures
Characterization of a dataset	Variability of atomic types	Low	High		
	Variability of molecules	Low		High	
	Variability of structure sources	Low			High
Reference to publication		[35] (set beg2)	[40]	–	–

We wanted to demonstrate that NEEMP is able to produce results comparable with previously published data. Therefore, we focused first on datasets for which EEM parameterization has been performed in the past. Specifically, our first two datasets—DTP_small and DTP_large (see Table 1) originate from the DTP NCI database [49] and were used in publications [35] and [40], respectively. In addition, we also wanted to provide new interesting and useful results for the research community. For this reason, we then focused on datasets of interest to bioinformatics and chemoinformatics, and which have never been subjected to EEM parameterization. Specifically, the next two datasets (CCD_gen and CCD_exp, see Table 1) were obtained from the wwPDB Chemical component dictionary (wwPDB CCD) database.

This database contains molecules which are parts of biomacromolecular structures deposited in Protein Data Bank [50]. Therefore, these molecules are highly biologically important and include drug molecules, metabolites, compounds from biochemical pathways, etc. For each molecule, the wwPDB CCD contains two types of coordinates, i.e, ideal coordinates generated by CORINA software [51] (included in our dataset CCD_gen) and model coordinates originating from experimental data (included in our dataset CCD_exp). wwPDB CCD is a database of “raw” structural data, therefore we had to perform several preprocessing steps to create our datasets. In this way we obtained the datasets CCD_gen_all and CCD_exp_all, which we used in the validation case study. But for our EEM parameterization goals, these datasets were too large (about four times larger than the dataset DTP_large). Therefore we reduced the size of datasets by a factor of four. Details about wwPDB CCD preprocessing and a summary of its results can be found in (Additional file 2) and (Additional file 3), respectively. Lists of the molecules in all datasets are in (Additional file 4).

The four datasets enable us to increase how demanding our EEM parameterization was in a stepwise manner and therefore show the strong and weak points of the LR and DE-MIN EEM parameterization approaches. The first dataset (DTP_small) is the easiest—small, with low variability of atomic types, molecules and structure sources. The second dataset (DTP_large) is more ambitious, because it is large and contains a large number of atomic types. The third dataset (CCD_gen) brings further complexity, since it contains heterogeneous types of molecules. The fourth dataset (CCD_exp) is the most challenging, because it has all the demands of CCD_gen and in addition, its structures originate from different experiments performed under highly varied conditions by various scientific teams.

*Selection and calculation of QM charges* The QM charge calculation approach B3LYP/6-311G/NPA was selected for calculating the QM charges used as inputs for the EEM parameterization. These charges were selected, because the B3LYP theory level, 6-311G basis set and NPA proved to be very suitable for EEM parameterization [37, 38, 40]. In addition, the same combination of B3LYP, 6-311G and NPA was used in publication [40], from which we took the dataset DTP_large. The QM charges were calculated by Gaussian [52] for all molecules from datasets DTP_small, DTP_large, CCD_gen and CCD_exp.

*EEM parameterization* The EEM parameterization was performed using NEEMP on all four prepared datasets and four different parameterization methodologies were used (LR with $R^2$ metrics, LR with *RMSD* metrics, DE-MIN with $R^2$ metrics and DE-MIN with *RMSD* metrics). Thus we obtained 16 EEM parameter sets, including their quality criteria. The molecules in all the datasets were not optimized before performing the EEM parameterization. This strategy was motivated by a fact, that the resulting EEM parameters should be utilized also for non optimized molecules, to keep the procedure of EEM charge calculation quick. The same strategy was successfully used in the past (e.g., in articles [9, 35, 40, 53]).

*Comparison of EEM parameterization methods LR and DE-MIN using metrics* $R^2$ and *RMSD*. The main quality criteria of the calculated EEM parameter sets are summarized in Table 2. Complete validation reports for all the EEM parameter sets are in (Additional file 5) and the particular EEM parameter sets are stored in (Additional file 6).

**Table 2:**
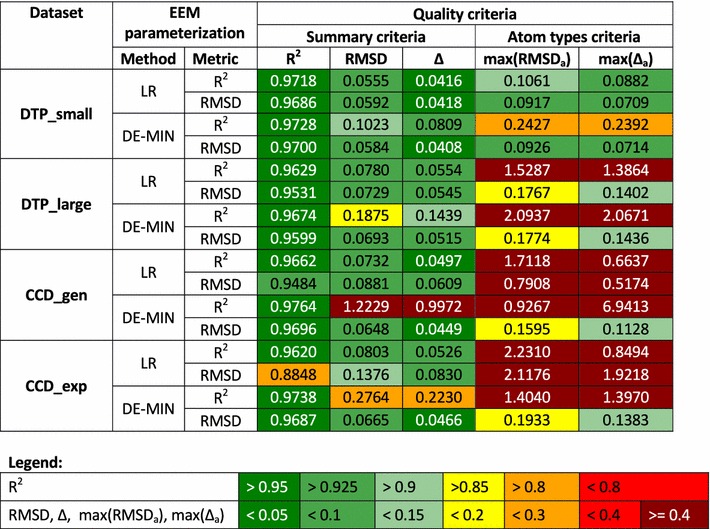
Quality criteria of EEM parameter sets calculated in parameterization comparison case study

For the simple dataset DTP_small, both LR and DE-MIN provide excellent results and both $R^2$ and *RMSD* metrics are applicable. Only the combination of DE-MIN with $R^2$ metrics performs slightly weaker.

For the bigger dataset DTP_large, which contains more atom types, differences between the tested approaches started to appear. Summary quality criteria are still very good for all of the approaches, but only the combinations LR+*RMSD* and DE-MIN+*RMSD* also have acceptable atom types criteria. Interestingly, the performance of LR+*RMSD* and DE-MIN+*RMSD* is still almost the same.

For the dataset CCD_gen, which brings a heterogeneity of molecules, the differences between the approaches markedly increase. LR still has good summary quality criteria, but the atom types quality criteria significantly worsen, even with LR+*RMSD*. Therefore, only the combination DE-MIN+*RMSD* seems to be applicable for this dataset and provides very good quality criteria.

**Fig. 2:**
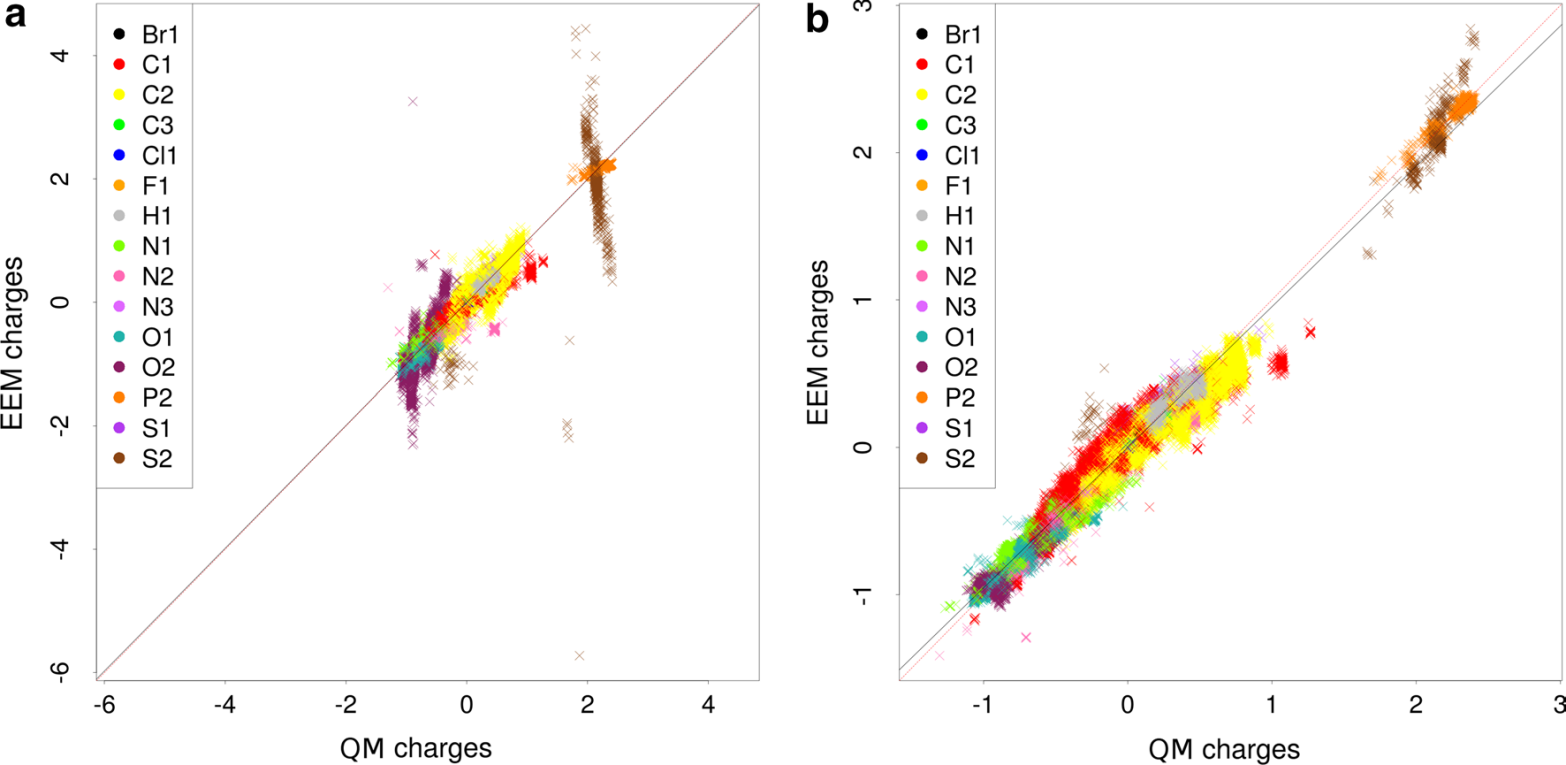
Graph of QM and EEM charges correlation for dataset CCD_exp and LR+*RMSD* approach (**a**) and DE-MIN+*RMSD* approach (**b**)

In the last and the most challenging dataset CCD_exp, the tested approaches demonstrate similar trends as for CCD_gen, but even more pronounced. LR also has weak summary quality criteria and the atom types quality criteria are highly problematic. Fortunately, the DE-MIN+*RMSD* approach is still applicable and provides quality criteria only slightly worse than for CCD_gen. A graph of the QM and EEM charges correlation for CCD_exp and the approaches LR+*RMSD* and DE-MIN+*RMSD* are shown in Fig. 2, and demonstrate that with such a large and heterogeneous dataset, the proper choice of EEM parameterization approach is crucial.

*Summary of comparison results* To conclude, we found that LR (with both metrics) is an appropriate approach for smaller and homogeneous datasets. On the other hand, DE-MIN (with *RMSD* metric) is a markedly more suitable solution for larger and more heterogeneous datasets.

#### Parameterization running time case study

The performance of NEEMP, measured on a standard personal computer is showed in Table 3.

**Table 3:** NEEMP performance on a standard personal computer (Intel i7-4790K CPU @ 4.00GHz)

Dataset	DTP_small		DTP_large		CCD_gen and CCD_exp	
EEM parameterization method	LR	DE-MIN	LR	DE-MIN	LR	DE-MIN
Running time	54 m	14 m	4 h 25 m	16 m	9 h 24m	25 m

All measurements were repeated 3 times, and we always considered the minimum running time of all the repetitions (in this way random interference of background activity of the operating system is masked out). Running time varies from a few minutes to several hours. As expected, there is no observable difference between CCD_gen and CCD_exp—the complexity depends on the number of molecules and atoms but not the specific values of atom coordinates or charges. In general, DE-MIN performs significantly better for all datasets. The difference becomes more apparent with a larger number of molecules, being caused by the discard algorithm, which has to examine more options (this step is not necessary for DE-MIN).

**Fig. 3:**
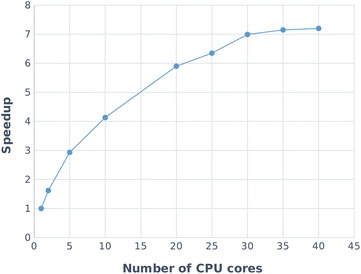
Speedup achieved by the parallel version run at different number of CPU cores

As described in the Implementation section, the code can run on multiple CPU cores in the heaviest computations, therefore the computation time can be markedly shortened. Figure 3 shows speedup of the parallel version on different number of CPU cores, i.e., how many times faster the parallel program runs compared to the single-core version. The experiments were run with the DE-MIN method and RMSD metric, on the CCD_exp dataset and using a machine with 4 Intel Xeon E7-4860 @ 2.27 GHz CPUs. Again, all measurements were repeated 3 times, and we always considered the minimum running time. The particular values of minimum running time are summarized in (Additional file 7: Table S2).

In the ideal case, if the workload was uniformly distributed among all the cores, the speedup would be the same as the number of cores. However, the measurement shows a decrease in efficiency of the parallel execution, which is a consequence of the non-uniform distribution of the workload (existence of non-parallel sections in fact). We can conclude that it is worth running NEEMP with **up to 20 CPU cores**, where we still get an approximately 6x speedup, but using more cores becomes a waste of resources. In general, with larger training sets, when there is more work to evaluate a single parameter vector, the efficiency will improve.

#### Parameterization calculation case study

*Goal* In this case study, we would like to obtain high-quality EEM parameters for the wwPDB CCD database and based on several frequently used QM charge calculation approaches. For this purpose, we will apply the knowledge obtained during our comparison of EEM parameterization approaches.

*Dataset preparation* During this comparison, we prepared two datasets for wwPDB CCD: CCD_gen and CCD_exp. CCD_gen provided EEM parameter sets with better quality criteria, therefore we will use this dataset.

*Selection and calculation of QM charges* The QM charge calculation approach B3LYP/6-311G/NPA was again selected—for the same reasons as in the parameterization comparison case study. Furthermore, B3LYP/6-311G/MPA was selected, because MPA is often used for EEM parameterization [31, 34–37] as well. Moreover, it was also used in combination with B3LYP/6-311G [40]. Then, the approaches B3LYP/6-311G*/NPA and B3LYP/6-311G*/MPA were selected. The reason for this was that the 6-311G* basis set had never been used for EEM parameterization, and EEM parameters for these approaches can be interesting and useful for the research community. The QM charges were calculated by Gaussian [52] for all molecules from the CCD_gen dataset, except for the B3LYP/6-311G/NPA charges, which were taken from the parameterization comparison case study.

*EEM parameterization* The EEM parameterization was performed by NEEMP on the CCD_gen dataset for the four above-mentioned QM charges. The DE-MIN+*RMSD* approach was used, because it provides the best results for CCD_gen in the parameterization comparison case study. Thus we obtained 4 EEM parameter sets, including their quality criteria.

**Table 4:**
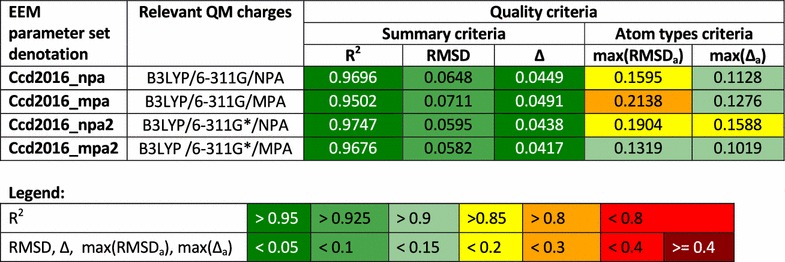
Denotations and main quality criteria of EEM parameter sets calculated in parameterization calculation case study

*Summary of EEM parameterization results* The denotations and the quality criteria of the obtained EEM parameter sets are summarized in Table 4. These results show that NEEMP provided us with four high-quality EEM parameter sets for the wwPDB CCD database. These EEM parameter sets are in (Additional file 6) and validation reports for them are in (Additional file 5).

### Validation case study

This case study first analyses the coverage of selected EEM parameter sets (Coverage validation case study) and then also the quality of these sets (Quality validation case study).

#### Coverage validation case study

*Goal* In this case study, we would like to compare the coverage of selected EEM parameter sets on key databases of small molecules. In this way, we introduce NEEMP functionality focused on the validation of coverage.

*EEM parameter sets* Several sets of published EEM parameter sets [34, 35, 37, 38, 40], i.e., the sets which proved to be of good quality in the past [10, 11, 40], and also four EEM parameter sets calculated in the parameterization calculation case study were selected for the coverage comparison. A list of the compared EEM parameter sets, including basic information about them, can be seen in the first three columns of (Additional file 8: Table S3).

*Databases* The coverage comparison was done on three well-known databases of biologically important small molecules: wwPDB CCD, DrugBank, and PubChem. The number of compounds in all these databases (from March 2016) are summarized in Table 5. wwPDB CCD, which was also used in the parameterization case study, is a medium-sized database including ligands incorporated in biomacromolecules. DrugBank is a relatively small database containing chemical compounds with medical applications. The PubChem database intends to include all common chemical substances, therefore it is very large and heterogeneous.

**Table 5:** Size of database, used for comparison of EEM parameter set coverages

Database	Number of compounds
DrugBank	7097
wwPDB CCD	21,741
PubChem	71,632,601

*Coverage comparison procedure* The coverage of all the tested EEM parameter sets was calculated via NEEMP for all three databases of interest. The results are summarized in (Additional file 8: Table S3).

*Summary of results* Interestingly, even though the databases are very different, the coverage values are very similar for all of them. Only the EEM parameter sets calculated recently (i.e., Cheminf2015 and Ccd2016 sets) exhibit sufficient coverage ($>93\,\%$ for all the databases). The other parameter sets have low coverage, specifically, they are only applicable for 40–80% of molecules from the tested databases. The coverage values for DrugBank and PubChem agree with information published in [40]. In general, this confirms that coverage is a weakness of the majority of currently published EEM parameter sets. We also showed that NEEMP enables us to easily obtain information about the EEM parameter set coverage for each database of interest.

#### Quality validation case study

*Goal* This case study compares the quality of selected EEM parameter sets on two datasets, which contain wwPDB CCD structures. It also shows NEEMP functionality focused on the validation of EEM parameter set quality.

*Preparation of datasets* Two datasets containing molecules from wwPDB CCD were used for quality comparison—a simple dataset for basic testing and a challenging dataset for deep analysis of EEM parameter set quality. The challenging dataset is specifically the dataset CCD_gen_all, which was prepared in the parameterization comparison case study and which includes structures generated by CORINA. This dataset contains all the wwPDB CCD molecules composed of atoms of C, H, N, O, S, P, F, Cl and Br and that have no structural errors. Therefore, it includes about 82 % of the whole of wwPDB CCD, it is highly chemically heterogeneous and demanding for calculating high-quality EEM charges. The simple dataset (denoted CCD_gen_CHNO) is a subset of CCD_gen_all. The list of molecules in this dataset can be found in (Additional file 4). This dataset was designed for a basic quality test of all the EEM parameter sets used in the coverage validation case study, and so it had to be completely covered by all these EEM parameter sets. For this reason, its molecules contain only the atoms C, H, N and O and do not include triple bonds. This fact implies its low chemical variability. Information about both datasets are summarized in Table 6.

**Table 6:** Description of datasets used in quality validation case study

	Datasets	
Designation	CCD_gen_CHNO*	CCD_gen_all*
Source database	wwPDB CCD	wwPDB CCD
Number of molecules	8144	17,769
Atomic types (elements and bond orders)	H1, C1, C2, N1, N2, O1, O2	H1, C1, C2, C3, N1, N2, N3, O1, O2, F1, P2, S1, S2, Cl1, Br1

*All other information about the dataset is the same as for the dataset CCD_gen, described in Table 1

*EEM parameter sets* The quality comparison was analyzed on the same EEM parameter sets as the coverage comparison. Specifically, when the quality comparison was performed on the dataset CCD_gen_CHNO, all these EEM parameter sets were used. The quality comparison on CCD_gen_all was only performed for the Cheminf2015 and Ccd2016 EEM parameter sets, because only these sets can be applied on all the molecules from this dataset.

*Calculation of QM charges* For molecules from the dataset CCD_gen_CHNO, we calculated the same charges as in the coverage validation case study, because EEM charges calculated using the tested EEM parameter sets had to be compared with corresponding QM charges. Therefore, the following QM charges were calculated: HF/STO-3G/MPA and NPA, B3LYP/6-31G*/MPA and NPA, B3LYP/6-311G/MPA and NPA, and B3LYP/6-311G*/MPA and NPA. For molecules from the dataset CCD_gen_all, we only calculated QM charges corresponding to the EEM parameter sets Cheminf2015 and Ccd2016. Therefore we calculated the QM charges B3LYP/6-311G/MPA and NPA, and B3LYP/6-311G*/MPA. The QM charges were calculated by Gaussian [52] or (where possible) taken from the parameterization case study.

**Fig. 4:**
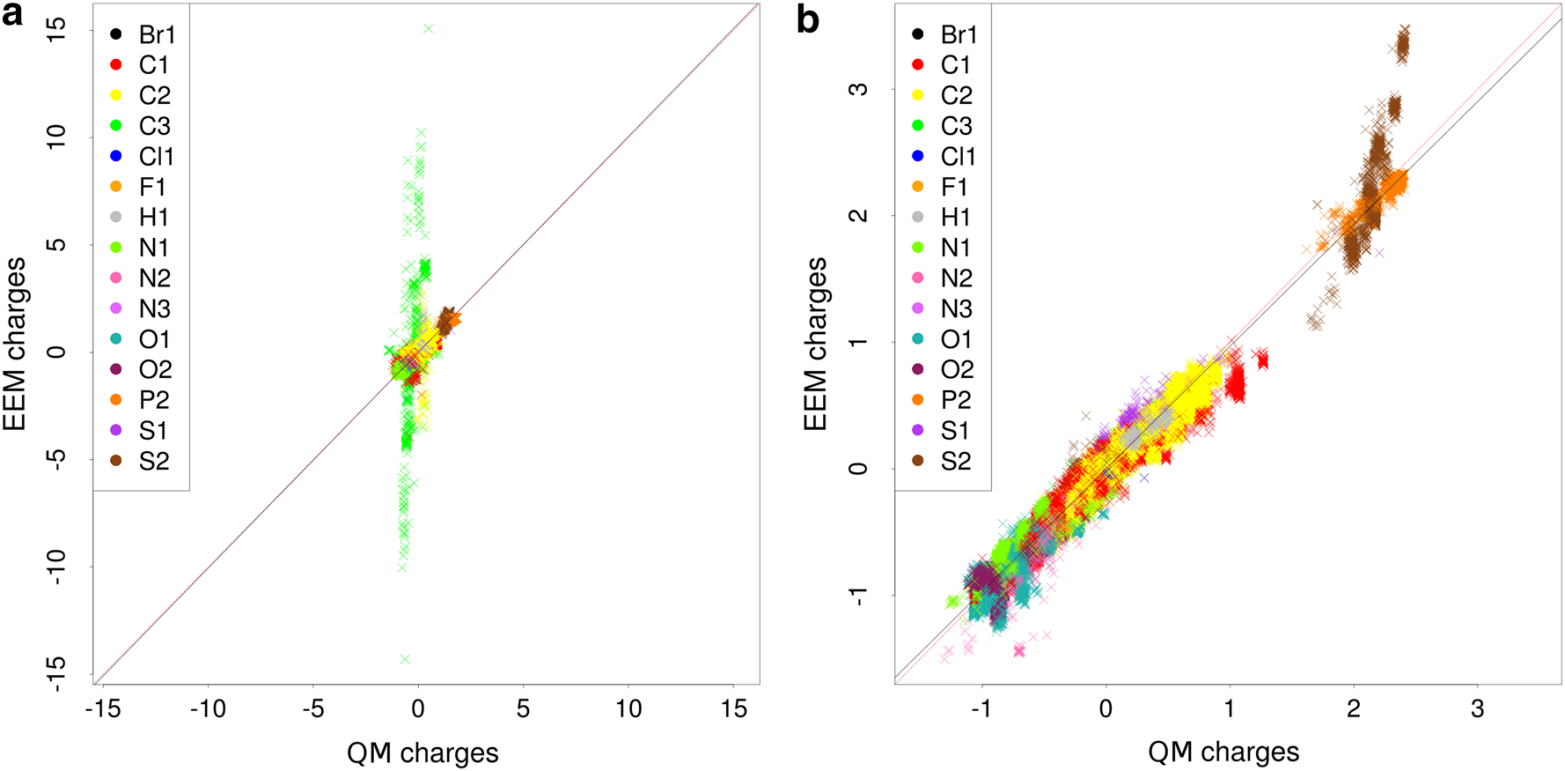
Graph of QM and EEM charges correlation for Cheminf2015_mpa parameter set (**a**) and Cheminf2015_npa parameter set (**b**) on the dataset CCD_gen_all. The graph for Cheminf2015_mpa includes a marked correlation problem at C3 atoms (they are in *green*), the graph for Cheminf2015_npa shows a slight correlation issue at S2 atoms (they are in *brown*)

*Quality comparison procedure* For the dataset CCD_gen_CHNO, the EEM charges were calculated using the same EEM parameter sets as in the coverage validation case study. Afterwards, these EEM charges were compared with the corresponding QM charges via NEEMP and the validation reports were created. A summary of the most important quality criteria and the validation reports are in (Additional file 9: Table S4) and (Additional file 5), respectively. For the dataset CCD_gen_all, the EEM charges were calculated using only the EEM parameter sets Cheminf2015 and Ccd2016. We then employed NEEMP to compare EEM charges with relevant QM charges and produce validation reports. The most important quality criteria are summarized in Table 7, selected correlation graphs are shown in Fig. 4 and all the validation reports are in (Additional file 5).

**Table 7:**
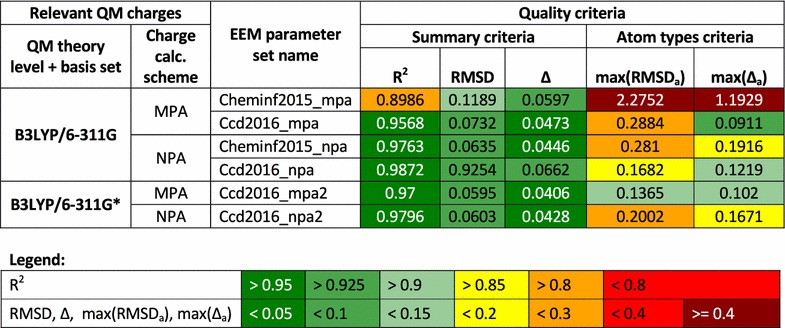
Quality criteria of the EEM parameter sets on the dataset CCD_gen_all

*Summary of results* All the EEM parameter sets proved to be of very high quality on the dataset CCD_gen_CHNO. Both the summary quality criteria and the atom type quality criteria were excellent. Specifically, $R^2$ was mostly higher than 0.95, $RMSD < 0.08$ and $max(RMSD_a) < 0.12$. This documents the fact that all these EEM parameter sets are very well adjusted for EEM charge calculation on datasets with low atom type variability. The results for the dataset CCD_gen_all are more heterogeneous (see Table 7). The summary criteria are excellent ($R^2 > 0.95$) or at least acceptable ($R^2 \sim 0.9$) for all the EEM parameter sets. But the atom type quality criteria are sometimes problematic. The Cheminf2015_mpa parameter set in particular produced very high $max(RMSD_a)$ and $max(\Delta _a)$ values. Figure 4a and the validation report shows that there is a problem with the correlation of charges on carbon atoms with triple bonds (C3 atoms). Further EEM parameter sets have sufficiently low atom type quality criteria. Two of the EEM parameter sets (Ccd2016_mpa and Cheminf2015_npa) contain slide correlation issues for S2 or C3—see an example in Fig. 4b. The remaining EEM parameter sets exhibited no problems or issues. Furthermore, the quality criteria of all Ccd2016 parameter sets are comparable to the quality criteria obtained during the calculation process (see Table 4). This fact confirmed the robustness of the EEM parameterization performed via NEEMP. In general, these results show that the datasets with high atom type variability can still represent a challenge for the available EEM parameter sets. Therefore, the EEM parameter set quality validation implemented in NEEMP is a very important step in EEM usage and application.

## Conclusion

We provide the software tool NEEMP, which offers three main functionalities: EEM parameterization (via the LR and DE-MIN method, with $R^2$ and *RMSD* metrics); EEM parameter set validation (i.e., validation of coverage and quality) and EEM charge calculation. NEEMP was implemented in C, contains parallelization and provides a fast and accurate solution for work with EEM. To the best of our knowledge, NEEMP is the only available software tool enabling EEM parameterization and EEM parameter set validation. In addition, the DE-MIN parameterization method is an innovative approach, developed by ourselves and first published in this work.

NEEMP functionality is demonstrated on two case studies—a parameterization and a validation case study.

The parameterization case study analyses the performance of both parameterization methods (LR and DE-MIN) and metrics ($R^2$ and *RMSD*) using four different datasets which increase the demands of EEM parameterization in a stepwise manner. We found that LR (with both metrics) is an appropriate approach for smaller and homogeneous datasets. On the other hand, DE-MIN (with *RMSD* metric) is a markedly more suitable solution for larger and more heterogeneous datasets. We also showed that NEEMP is able to perform EEM parameterizations in a reasonable time, and its execution on multiple processors produces a marked speedup. We then performed EEM parameterization via the DE-MIN method with *RMSD* metrics on wwPDB CCD—a database of ligands found in biomacromolecular structures. This database is frequently used by the life science community and it has never been subjected to EEM parameterization. Despite the high heterogeneity of the database, we produced 4 high-quality EEM parameter sets. This demonstrated, that NEEMP is highly applicable for the computation of new EEM parameter sets.

The validation case study focused first on coverage validation. Specifically, we validated the coverage of selected EEM parameter sets (i.e., several published EEM parameter sets and EEM parameter sets provided in this article) on three well-known databases of small molecules (wwPDB CCD, PubChem and DrugBank). It was shown that the coverage of older EEM parameter sets is problematic. Specifically, they are only applicable for 40–80 % of molecules from the tested databases. Only the recently published Cheminf2015 EEM parameter sets and the EEM parameter sets provided in this article had sufficient coverage (>90–95 %). The case study then also focused on quality validation of the selected EEM parameter sets. All the sets performed very well on a small dataset with molecules comprised of C, H, N and O. On the other hand, the larger and more heterogeneous dataset (17,769 molecules; 15 atom types) was a challenge for most of the tested EEM parameter sets. The older parameter sets could not cover the dataset and the newer ones (i.e., Cheminf 2015) had accuracy problems with some atom types. The only applicable EEM parameter sets were the Ccd2016 sets provided in this article. From these results it can be seen that EEM parameter set coverage and quality can still be problematic. Therefore it makes sense to verify the coverage and quality of EEM parameter sets before their use, and NEEMP is an appropriate tool for such verification.

Moreover, from both case studies it seems that it is still necessary to perform new EEM parameterizations and obtain EEM parameter sets with high quality and coverage on key structural databases.

Last but not least, NEEMP can potentially help the community to perform EEM parameterizations which are challenging. For example, EEM parameterization based on HF/6-31G*/MK QM charges. Mimicking these QM charges via EEM is very important because they are used for AMBER partial-charge parameterization routine focused on biomolecular ligands [54–56]. On the other hand, EEM is documented as an approach which performs very weakly for MK charge calculation scheme [10, 37, 40]. Employing NEEMP can help us to override the problems with MK based EEM parameterizations, or it can confirm limitations of EEM in this domain. Further challenging EEM parameterizations, which can be potentially solved via NEEMP, are parameterizations focused on proteins or metalloproteins—large macromolecules containing long-range interactions.

## Availability and requirements

**Project name:** NEEMP

**Project home page:** http://ncbr.muni.cz/neemp

**Operating system(s):** Linux (recommended), Windows, Mac OS X

**Programming language:** C, external library in Fortran

**Other requirements:** GNU Fortran, libxml2, LAPACK, zlib, OpenMP

**License:** GNU GPLv3

**Any restrictions on use by non-academics:** no restrictions

## Additional files

10.1186/s13321-016-0171-1 Source codes. Archive with source codes of NEEMP.

10.1186/s13321-016-0171-1 Preprocessing information. Detailed description of wwPDB CCD preprocessing.

10.1186/s13321-016-0171-1 Summarization of preprocessing results for individual molecules from wwPDB CCD.

10.1186/s13321-016-0171-1 List of molecules. Lists of molecules in all datasets.

10.1186/s13321-016-0171-1 Validation reports. Validation reports for EEM parameter sets, calculated in parameterization case study and for EEM parameter sets, tested in validation case study.

10.1186/s13321-016-0171-1 EEM parameter sets. EEM parameter sets, calculated in the parameterization case study.

10.1186/s13321-016-0171-1 NEEMP running times on more CPUs.

10.1186/s13321-016-0171-1 Summary information about coverage of tested EEM parameter sets, performed on databases wwPDB CCD, DrugBank and PubChem.

10.1186/s13321-016-0171-1 Quality criteria of the EEM parameter sets on the dataset CCD_gen_CHNO.
