# Supplementary material for: NEEMP: software for validation, accurate calculation and fast parameterization of EEM charges
Source: J Cheminform. 2016 Oct 17;8:57. doi: 10.1186/s13321-016-0171-1 (PMC5067907; doi:10.1186/s13321-016-0171-1)
Supplement: Supplementary file 2 — 10.1186/s13321-016-0171-1 Preprocessing information. Detailed description of wwPDB CCD preprocessing. [file 13321_2016_171_MOESM2_ESM.pdf]

Detailed description of wwPDB CCD preprocessing

The preprocessing was performed on the complete wwPDB CCD database (downloaded in March 2016), which contained 21,741 molecules.

It consisted of four steps: Removing positively or negatively charged molecules, selecting of molecules with relevant atom types, validating molecular structures and removing problematic molecules, and limiting the dataset size.

The first step – removing charged molecules - was performed because previous EEM parameterizations were also only done on neutral molecules.

In the second step, we only selected molecules composed of the same atom types as the data set DTP_large. The only exceptions were I1 and P1, which could not be included into the CCD_* datasets, because they did not occur in wwPDB CCD often enough, therefore their number was not sufficient for robust EEM parameterization. Specifically, they occurred only 241 times (I1) and 35 times (P1). The other atomic types had more than 500 occurrences.

As a third step, we performed a validation of molecular structures and we examined whether the basic structure quality criteria were fulfilled. Namely, we checked whether the molecules contained atom clashes (i.e., two atoms too close to each other), bonds that were too long and atoms with the wrong number of bonds (e.g., a carbon with only three single bonds). At this stage also obsolete ligands, ligands containing atoms with missing coordinates, ligands with only a 2D structure and ligands that were too small (no atoms, 1 atom, 2 atoms of the same element) were removed. In this way we obtained the datasets CCD_gen_all and CCD_exp_all, containing 17,769 molecules. These datasets were used in the second case study, focused on validation. But for our EEM parameterization goals, these datasets were too large (about four times larger than the dataset DTP_large).

Therefore, in the fourth step, we reduced the size of datasets by a factor of four. This was done by including every fourth molecule (the first, the fifth, the ninth etc.). Thus, we obtained the datasets CCD_gen and CCD_exp, containing 4,443 molecules.
