# Supplementary material for: NEEMP: software for validation, accurate calculation and fast parameterization of EEM charges
Source: J Cheminform. 2016 Oct 17;8:57. doi: 10.1186/s13321-016-0171-1 (PMC5067907; doi:10.1186/s13321-016-0171-1)
Supplement: Supplementary file 7 — 10.1186/s13321-016-0171-1 NEEMP running times on more CPUs. [file 13321_2016_171_MOESM7_ESM.pdf]

Number of cores	1	2	5	10	20
**Efficiency**	1,00	0,81	0,59	0,41	0,30
**Speedup**	1,00	1,62	2,93	4,13	5,90
**Runninng time (s)**	13524	8348	4611	3272	2292

25	30	35	40
0,25	0,23	0,20	0,18
6,35	6,99	7,15	7,20
2130	1934	1892	1878
