# Supplementary material for: NEEMP: software for validation, accurate calculation and fast parameterization of EEM charges
Source: J Cheminform. 2016 Oct 17;8:57. doi: 10.1186/s13321-016-0171-1 (PMC5067907; doi:10.1186/s13321-016-0171-1)
Supplement: Supplementary file 8 — 10.1186/s13321-016-0171-1 Summary information about coverage of tested EEM parameter sets, performed on databases wwPDB CCD, DrugBank and PubChem. [file 13321_2016_171_MOESM8_ESM.pdf]

Relevant QM charges		EEM parameter set name	Summary	
QM theory level + basis set	Charge calc. scheme		R^2	RMSD
HF/STO-3G	MPA	Baek1991	0.9157	0.065
		Svob2007_cbeg2	0.9779	0.0254
		Svob2007_chal2	0.9711	0.0283
		Svob2007_chm2	0.9673	0.0292
		Svob2007_cmet2	0.9722	0.0303
B3LYP/6-31G*	MPA	Bult2002_mpa	0.9687	0.058
		Bult2002_npa	0.9703	0.0675
	NPA	Ouy2009	0.9636	0.0744
		Ouy2009_elem	0.9657	0.0723
B3LYP/6-311G	MPA	Cheminf2015_mpa	0.9561	0.0701
		**Ccd2016_mpa**	0.9727	0.0583
	NPA	Cheminf2015_npa	0.9617	0.0586
		**Ccd2016_npa**	0.968	0.0632
B3LYP/6-311G*	MPA	**Ccd2016_mpa2**	0.9738	0.0517
	NPA	**Ccd2016_npa2**	0.9744	0.0562

Quality		
	Atom types	
Δ	max($RMSD_a$)	max(Δ_a)
0.0543	0.0814	0.0655
0.0213	0.0526	**0.037**
0.0237	**0.0488**	**0.0346**
0.0241	0.0539	**0.0482**
0.0261	0.0539	**0.0482**
0.0423	0.0988	0.0773
0.0483	0.1185	0.0959
0.0525	0.1932	0.1675
0.0506	0.1239	0.103
0.0539	0.1144	0.0988
0.0434	0.0956	0.0711
0.0411	0.0941	0.0716
0.0442	0.0993	0.0678
0.0378	0.0727	0.0576
0.0417	0.0963	0.0777
